# HSPA9/Mortalin mediates axo-protection and modulates mitochondrial dynamics in neurons

**DOI:** 10.1038/s41598-021-97162-1

**Published:** 2021-09-06

**Authors:** Cécile A. Ferré, Anne Thouard, Alexandre Bétourné, Anne-Louise Le Dorze, Pascale Belenguer, Marie-Christine Miquel, Jean-Michel Peyrin, Daniel Gonzalez-Dunia, Marion Szelechowski

**Affiliations:** 1 Toulouse Institute for Infectious and Inflamatory Diseases (Infinity), Université Toulouse, CNRS, Inserm, UPS, Toulouse, France; 2 Centre de Recherches sur la Cognition Animale (CRCA), Centre de Biologie Intégrative (CBI), Université de Toulouse, CNRS, UPS, Toulouse, France; 3 Sorbonne Universités, Faculté des Sciences et Technologie, CNRS UMR 8246, INSERM U1130, Neurosciences Paris Seine, Institut de Biologie Paris Seine, Paris, France

**Keywords:** Neurodegeneration, Mitochondria

## Abstract

Mortalin is a mitochondrial chaperone protein involved in quality control of proteins imported into the mitochondrial matrix, which was recently described as a sensor of neuronal stress. Mortalin is down-regulated in neurons of patients with neurodegenerative diseases and levels of Mortalin expression are correlated with neuronal fate in animal models of Alzheimer's disease or cerebral ischemia. To date, however, the links between Mortalin levels, its impact on mitochondrial function and morphology and, ultimately, the initiation of neurodegeneration, are still unclear. In the present study, we used lentiviral vectors to over- or under-express Mortalin in primary neuronal cultures. We first analyzed the early events of neurodegeneration in the axonal compartment, using oriented neuronal cultures grown in microfluidic-based devices. We observed that Mortalin down-regulation induced mitochondrial fragmentation and axonal damage, whereas its over-expression conferred protection against axonal degeneration mediated by rotenone exposure. We next demonstrated that Mortalin levels modulated mitochondrial morphology by acting on DRP1 phosphorylation, thereby further illustrating the crucial implication of mitochondrial dynamics on neuronal fate in degenerative diseases.

## Introduction

Neurodegeneration is a pathological phenomenon resulting from a complex interaction between environmental factors and genetic predispositions, occasioning the death of specific subpopulations of neurons. Nerve terminals are affected before neuronal cell bodies, which leads to neuronal disconnection and synapse impairment that may contribute to the early cognitive symptoms in patients^1^. However, the modalities of the retraction process of axons and its implication in the propagation of the degenerative signal, called *dying back*, remain elusive and seem to significantly differ from those involved in conventional apoptosis^2,3^. Indeed, axons and somas react very differently to stress and axons have higher protective signaling capacities than neuronal cell bodies. Insights have been brought by the analysis of Wallerian degeneration^4,5^, which enlightened the central role of mitochondria and mitochondrial dynamics in the synaptic and axonal dysfunctions that mark the initiation of neurodegeneration^6–8^. Moreover, we recently established a strong association between mitochondrial fragmentation, axonal vulnerability and retrograde spreading of degenerative processes^7^. Accordingly, we also demonstrated the neuroprotective ability of a viral protein, the X protein of Borna disease virus, which increases mitochondrial filamentation, thereby preserving mitochondrial function in the axonal compartment of stressed neurons and promoting neuronal survival in models of Parkinson’s disease (PD)^9,10^. Consequently, manipulating mitochondrial morphology and dysfunction in axons might have broad therapeutic implications, especially for neurodegenerative diseases^11,12^.

Mortalin (or Hspa9, Grp75, PBP74, mtHsp70) is a mitochondrial chaperone belonging to the heat shock 70 kDa protein family, which has been closely associated with mitochondrial sensitivity to stress in degenerative neurons^13^. Mortalin was originally described as an actor of protein translocation into the matrix^14,15^. Mortalin was next related to sensing of oxidative stress and activation of antioxidant pathways^16^, thereby conferring resistance against oxidative stress-induced apoptosis^17^, as well as against in vitro neurotoxicity, including models of ischemia, synucleinopathy or amyloid β (Aβ _1–42_) toxicity^18–20^. The neuroprotective properties of Mortalin were shown to rely on its ability to preserve the mitochondrial membrane potential and to protect from the accumulation of reactive oxygen species and the peroxidation of lipids^21,22^. The protein amount of Mortalin is thought to be of critical importance for neuronal fate, since decreased levels of Mortalin were found in brains of PD^23,24^ or Alzheimer’s disease patients^25^. However, the link between Mortalin gene mutations and risk for neurodegenerative diseases is still unclear^26–28^. Interestingly, the impact of Mortalin levels on neuronal fate seems to be closely related to mitochondrial function and morphology. Indeed, Mortalin down-regulation decreases mitochondrial respiration^29^ and produces a drop of mitochondrial targeting to synapses^30^. It also worsens Aβ-induced mitochondrial fragmentation and decreases ATP production^25^. In contrast, Mortalin overexpression was shown to be protective *in vitro*^21,22^, leading to extended cellular lifespan, from nematodes to humans^31,32^. However, the connections between Mortalin levels, mitochondrial physiology and neuronal fate during neurodegeneration are still missing, especially in the distal axonal compartment.

In this study, we sought to improve our knowledge on both the neuroprotective ability of Mortalin as well as on the intrinsic mechanisms of neuronal degeneration. We first observed that mitochondrial stress induced down-regulation of Mortalin in primary cultures of cortical neurons. To assess the impact of the variation of Mortalin levels on axonal susceptibility to mitochondrial stress, we used oriented neuronal cultures grown in microfluidic-based devices, which allow a physical separation of axons from their cell bodies and we mimicked the first steps of neurodegeneration by inducing a distal oxidative stress^6,7,10^. We used lentiviral vectors to modulate Mortalin levels and we analyzed the impact on mitochondrial morphology, dynamics and sensitivity to distal oxidative stress.

## Results

### Mortalin expression levels correlate with neuronal fate upon treatment with rotenone

**Figure 1:**
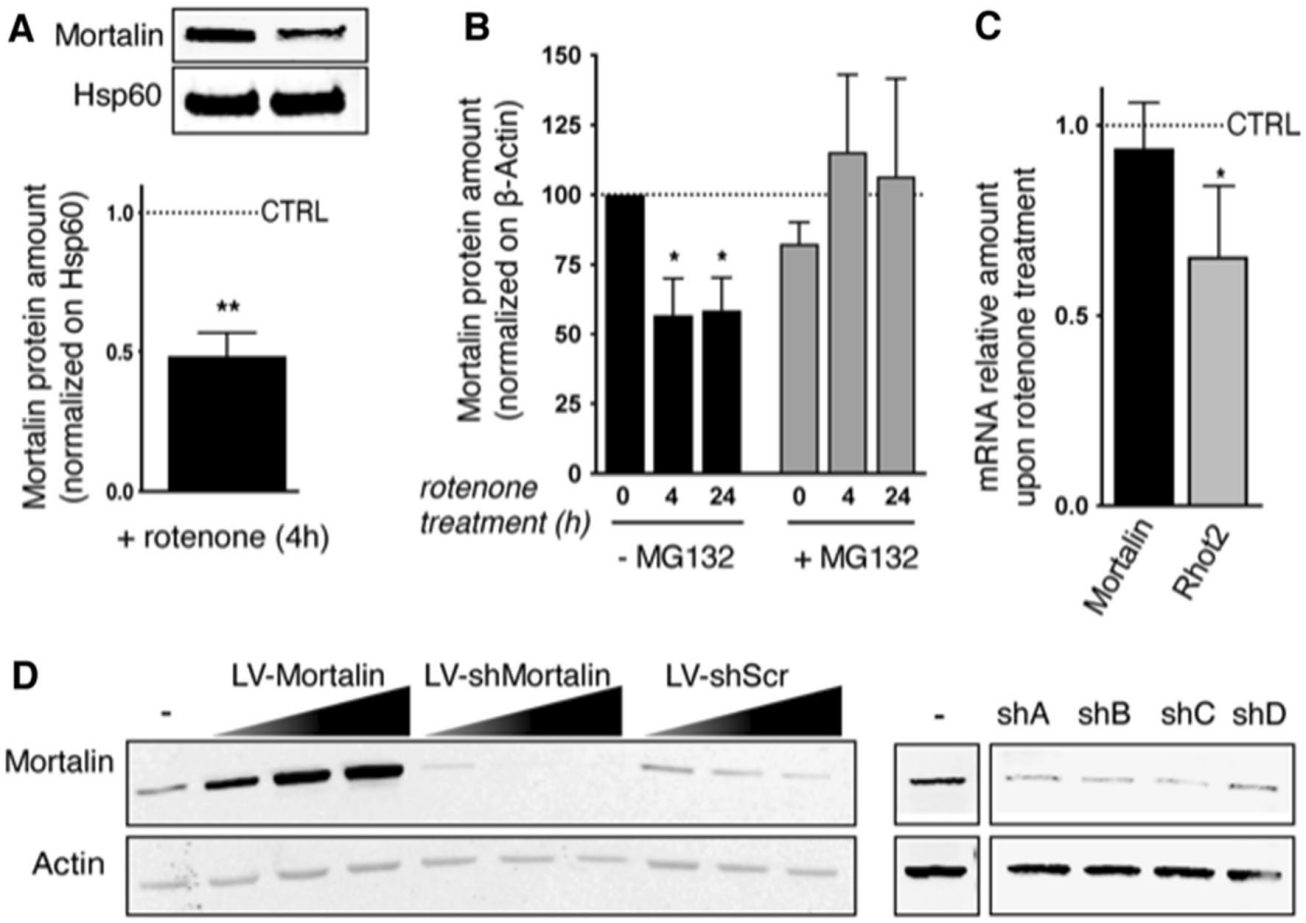
Modulation of Mortalin levels in primary cultured neurons. (**A**) Mortalin is down-regulated in neurons upon treatment with rotenone. Rat embryonic primary cortical neurons (DIV 12) were treated or not with 10 nM rotenone (+ rotenone) for 4 h and protein extracts were prepared from mitochondria-enriched fractions. Mortalin amounts were assessed by Western blot, using the mitochondrial chaperone Hsp60 or ß-Actin as loading controls. The graph represents the normalized ratio relative to untreated neurons and is expressed as the mean ± SD of 4 independent experiments. (**B**) Mortalin loss upon rotenone treatment is due to proteasomal degradation. Neurons (DIV 12) were treated or not with 10 nM rotenone for 4 or 24 h with or without MG132. Neuronal extracts were then analyzed by Western blotting for Mortalin protein quantification. ß-Actin was used as a loading control. The graph represents means ± SD of Mortalin amounts in treated neurons, normalized on control, untreated ones (100%), in 3 independent experiments. (**C**) Mortalin RNA levels are not affected by rotenone treatment. RNA was extracted from neurons treated or not with 10 nM rotenone for 4 h. qRT-PCR experiments were performed to determine mRNA levels for Mortalin and Rhot2 (Miro2 gene), as well as for Mdh1, a housekeeping gene. The ratios of Mortalin and Rhot2 mRNA levels (normalized on Mdh1) in rotenone treated *vs*. untreated neurons were calculated and data are presented as means ± SD of 4 independent experiments. (**D**) Modulation of Mortalin expression by transduction with lentiviral vectors (LV). Overexpression was achieved with a LV bearing an expression cassette for the rat Mortalin gene. Mortalin down-regulation was driven by cell transduction with lentiviral vectors (numbered sh **A** to **D**) expressing a set of specific 29-mer shRNA directed against Mortalin (shMortalin), or a control scramble sequence (shScr, Origene TR704140). Different multiplicities of transduction (1, 2, 4) were used to test the percentage of over- or under-expression. WB are presented as cropped parts of full blots displayed as supplemental information. DIV: days in vitro. *: *p* < 0.05, **p < 0.01, by Mann–Whitney non parametric t-test.

**Figure 2:**
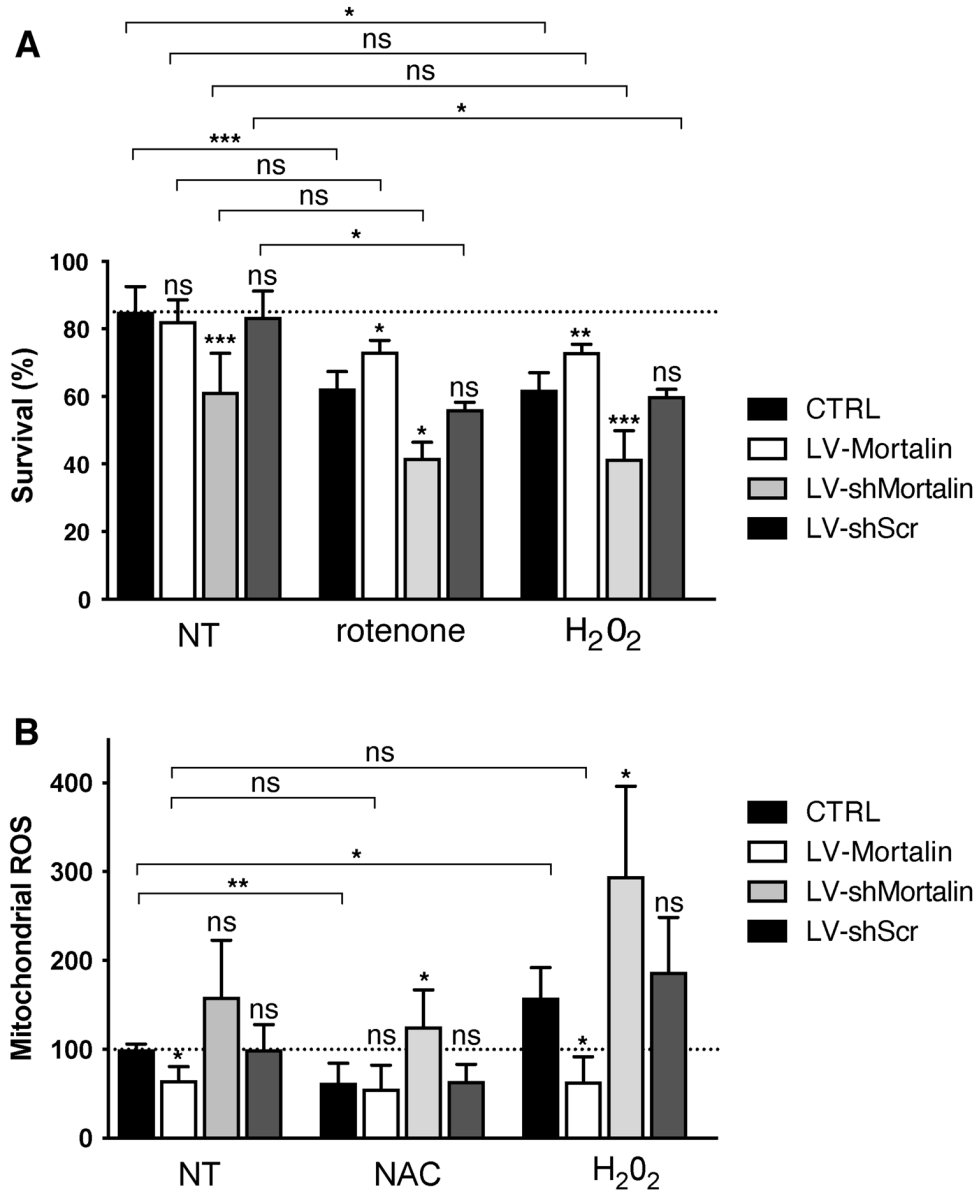
Mortalin levels control neuronal survival against stress induced by rotenone and H_2_O_2_ and mitochondrial accumulation of ROS. Rat embryonic primary cortical neurons were transduced on DIV 3 with lentiviral vectors (LV) to induce over- (+ 100%, LV-Mortalin) or under- (− 50%, LV-shMortalin, clone **A**) expression of Mortalin. A lentiviral vector expressing a shRNA with a scramble sequence was used as a control (LV-shScr). (**A**) On DIV 11, neurons were treated or not with 10 nM rotenone for 24 h, or with hydrogen peroxide (H_2_O_2,_ 100 μM) and then processed the day after. Percentages of cells showing pyknosis (revealed by DAPI staining) are presented as means ± SD of 4 independent experiments. (**B**) On DIV 11, neurons were treated or not with 100 μM NAC or H_2_O_2_. After 24 h, mitochondrial ROS accumulation was measured using the MitoSOX dye. Values were normalized using fluorometric values obtained for untreated and non-transduced neurons for each experiment. Results are presented as means ± SD of 3 independent experiments. ****p < 10^−4^, by 1-way ANOVA with Sidak’s multiple comparison *post-hoc* test.

In PD, Mortalin expression levels are strongly correlated to neuronal degeneration^13,20,23,24,33^. Considering the importance of defects in mitochondrial respiratory complex I activity, both in the human disease and in animal models of PD^34–36^, we first examined expression levels of Mortalin in rat primary cortical neurons treated with rotenone, a lipophilic cell-permeable toxin of complex I that, among other functions, induces ROS overproduction and oxidative stress, leading to PD symptoms^37^. Interestingly, we observed that treating neurons with 10 nM rotenone for 4 h was sufficient to decrease Mortalin expression by roughly 50% (Fig. 1A, Sup Fig. 1). Interestingly, inhibiting proteasome activity with MG132 prevented the Mortalin down-regulation triggered by rotenone (Fig. 1B). In contrast, Mortalin mRNA levels were not affected by rotenone, whereas mRNA levels of Rhot2, a gene implicated in the apoptosis pathways and known to be targeted by rotenone were decreased as expected^38^ (Fig. 1C). Altogether, our results suggest that rotenone treatment induces Mortalin degradation by the proteasome. To further establish the pathophysiological link between Mortalin expression levels and neuronal death induced by oxidative stress, we modulated Mortalin amounts in primary cortical neurons and we analyzed neuronal outcome after rotenone treatment. This was achieved by transducing neurons with lentiviral vectors expressing either rat Mortalin (LV-Mortalin), or a set of 4 different shRNA directed against rat Mortalin transcript (LV-shMortalin, numbered A to D) to over- or under-express Mortalin, respectively (Fig. 1D). All shRNA displayed comparable efficacies to down-modulate Mortalin expression and using combinations of several of these at the same time was not particularly more effective (Sup Fig. 2). Since a 50% decrease of Mortalin expression was observed in stressed neurons, we used titers of LV preparations for one shRNA (shA) sufficient to roughly double or halve Mortalin protein amount when compared to non-transduced neurons (Sup Fig. 3). A lentiviral vector expressing a control shRNA (scrambled sequence of the shA, LV-shScr) was used as a control of Mortalin silencing (Sup Fig. 3). Transduced neurons were grown for 11 days in vitro (DIV) and then treated or not with rotenone (10 nM) for 24 h, or with H_2_O_2_ for 30 min. Neurons were then fixed and neuronal health was appraised by analyzing nuclear pyknosis (Fig. 2A). Interestingly, in basal conditions, neurons under-expressing Mortalin were more pyknotic than control ones and displayed only 63% of healthy neurons, as compared to 85% for control neurons and 80% in neurons overexpressing Mortalin. After rotenone treatment, cultures displayed a 25% drop in healthy neurons, with the notable exception of neurons overexpressing Mortalin, which were protected from rotenone insult. Strikingly, the same results were obtained when using H_2_O_2_ (Fig. 2A). We next sought to directly measure the impact of Mortalin modulation on ROS levels. To this aim, we used the MitoSOX dye, a fluorescent indicator of mitochondrial ROS. We observed that mitochondrial ROS levels were inversely correlated with Mortalin amounts in basal conditions (Fig. 2B). Moreover, whereas control or Mortalin under-expressing neurons responded to treatments with H_2_O_2_ or NAC by respectively increasing or decreasing ROS levels, neurons overexpressing Mortalin displayed comparable levels of mitochondrial ROS whatever the treatment, suggesting a protection against ROS accumulation (Fig. 2B). This protection was also evidenced when measuring mitochondrial membrane potential, as Mortalin overexpression protected from rotenone-induced mitochondrial failure (Sup Fig. 4). Hence, Mortalin expression levels are directly correlated with both neuronal health and the ability to resist to oxidative stress.

### Mortalin expression levels drive axonal sensitivity to mitochondrial stress

**Figure 3:**
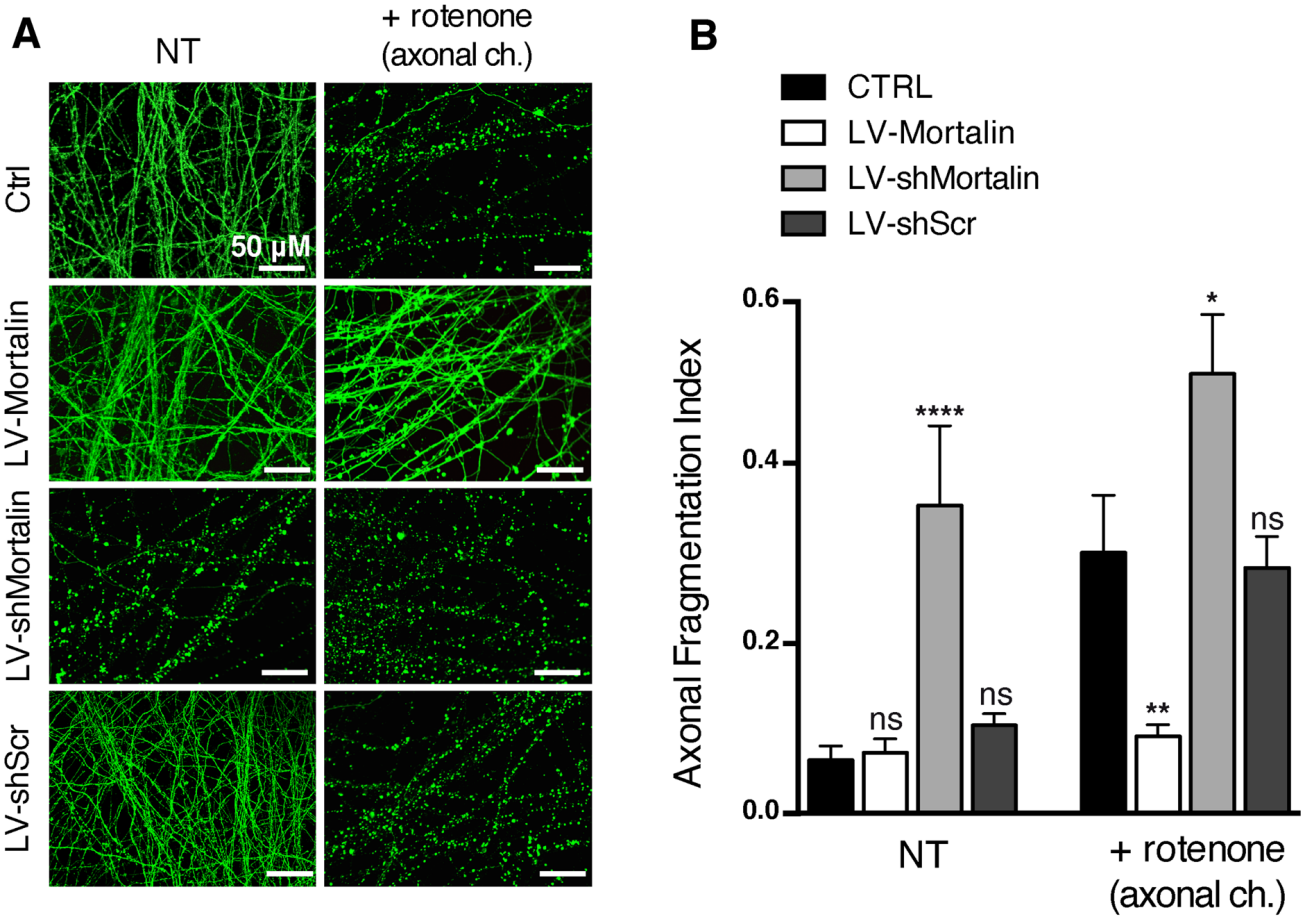
Mortalin expression controls axonal fate against a distal oxidative stress insult. Rat embryonic primary cortical neurons were grown in microfluidic devices for 12 DIV. Neurons were transduced on DIV 3 with lentiviral vectors (LV) to induce over- (LV-Mortalin) or under- (LV-shMortalin, clone **A**) expression of Mortalin. A lentiviral vector expressing a shRNA with scramble nucleotide sequence was used as a control (LV-shScr). On DIV 11, neurons were treated or not with 1 μM rotenone applied in the axonal chamber (+ rotenone) for 24 h and fixed on DIV 12. (**A**) Pictures of βIII-tubulin staining were randomly taken within axonal chambers (4 by condition) and the axonal fragmentation indexes were measured for each picture as the number of axonal dots per unit of tubulin staining. (**B**) Results are presented as means ± SD of 4 to 10 independent microfluidic devices, from 4 independent neuronal cultures. *p < 0.05, **p < 0.01, ****p < 10^−4^, by 1-way ANOVA with Sidak’s multiple comparison *post-hoc* test.

Mitochondrial dysfunction and oxidative stress occur early and distally in the pathological process of neurodegeneration. We previously showed that mitochondrial physiology strongly gates axonal vulnerability towards direct axonal insults and that rotenone needs to be used at high doses to trigger direct axonal degeneration^7^. Thus, we addressed the influence of Mortalin expression on axonal integrity and sensitivity to rotenone, using rat primary cortical neurons grown in microfluidic devices and transduced with LV-Mort, LV-shMortalin or LV-shScr. Mature and transduced neurons were then subjected (or not) to a strong treatment with 1 μM rotenone in low glucose medium, applied in the axonal chamber for 16 h. Indeed, we previously showed that somas and axons behave differently when treated with rotenone: this treatment applied in the axonal chamber induces axonal fragmentation through mitochondrial dysfunction, without any overt sign of apoptosis in the somatic chamber^6,10,39^. In contrast, the same treatment applied in the somatic chamber induces massive apoptosis of the whole culture and the axons appear completely fragmented (pyknotic nuclei and index of fragmentation reaching about 0.4, as shown in Sup Fig. 5). After 16 h of axonal treatment, neurons were fixed and stained for DAPI and βIII-tubulin, to evaluate, respectively, nuclear and axonal integrity (Fig. 3A). Consistent with our previous findings, axons of non-transduced neurons presented severe fragmentation after rotenone treatment (mean fragmentation index of 0.31 as compared to 0.06 for untreated, Fig. 3B). Interestingly, neurons under-expressing Mortalin exhibited strong baseline levels of axonal fragmentation (0.36), which was further enhanced after rotenone treatment (0.52), reaching levels observed when rotenone was added directly in the somatic chamber. In sharp contrast, neurons over-expressing Mortalin were almost completely protected from rotenone insult (Fig. 3), since they presented a very slight and non-significant increase of fragmentation when treated with rotenone (0.09 *versus* 0.07 without rotenone). Hence, Mortalin expression levels in neurons have a direct impact on the axonal sensitivity to rotenone.

### Mitochondria morphology and dynamics are modulated by Mortalin expression levels in neurons

**Figure 4:**
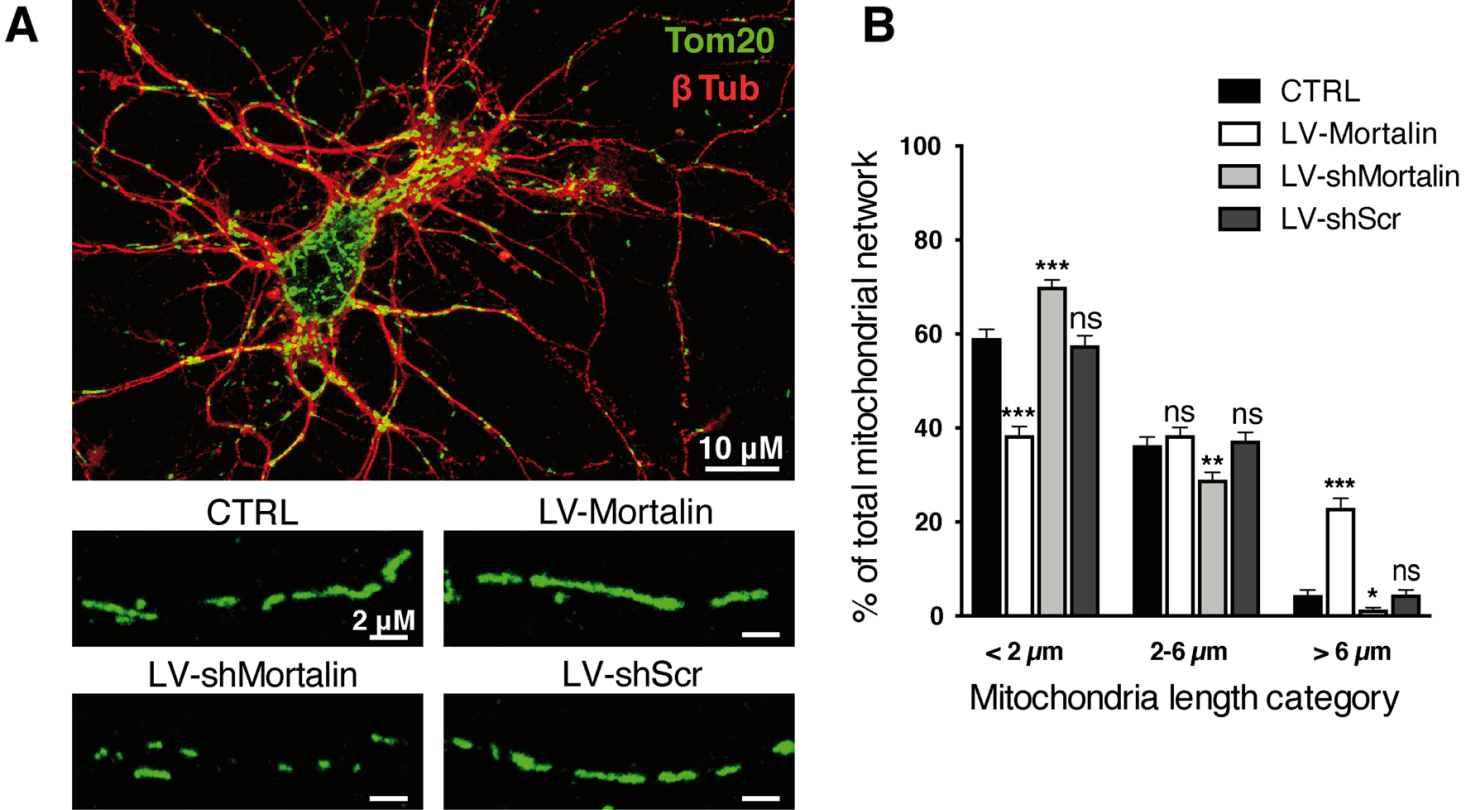
Mortalin modulates mitochondrial morphology in neuronal extensions. Rat embryonic primary cortical neurons were transduced on DIV 3 with lentiviral vectors (LV) to induce over- (LV-Mortalin) or under- (-LV-shMortalin, clone **A**) expression of Mortalin. A lentiviral vector expressing a shRNA with a scramble sequence was used as a control (LV-shScr). (**A**) Neurons were fixed on DIV 12 and both neuronal and mitochondrial networks were revealed by staining with, respectively, Tom20 and βIII-tubulin (βIII-Tub). Pictures were randomly taken and mitochondrial lengths were measured in all neuronal extensions. (**B**) For each neuron, the sum of the lengths of all measured mitochondria was calculated (mitochondrial network), within which the relative proportions of short (< 2 m), medium (2–6 m) and long (> 6 m) mitochondria were considered. The graph represents means ± SD of 18 to 40 neuronal mitochondrial networks, from 4 independent neuronal preparations. *p < 0.05, **p < 0.01, ***p < 0.001, by 2-way ANOVA, Kruskal Wallis test and Dunn’s multiple comparisons as *post-hoc* tests.

Previously, Park et al*.* documented that Mortalin downregulation exacerbates mitochondrial fragmentation induced by Aβ(1–42)^25^. We also showed that mitochondrial fragmentation was correlated with axonal vulnerability and the ability to spread “death signals”^7^. We therefore assessed the impact of modulating Mortalin expression levels on mitochondrial morphology, as well as on rotenone-induced mitochondrial fission in axons. To this aim, we performed immunofluorescence assays with Tom20, to reveal the mitochodrial network of primary cortical neurons transduced with LV-Mortalin or LV-shMortalin (Fig. 4A) and measured the lengths of mitochondria within neurites (Fig. 4B). In untreated neurons, mitochondrial length was unevenly distributed in three categories, from short (< 2 μm, 60% of total network), intermediate (2–6 μm, 35%) and long (> 6 μm, 5%) mitochondria. Interestingly, Mortalin down-regulation enhanced the proportion of short mitochondria (70%) to the detriment of intermediate and long mitochondria (respectively, 29% and 1%). Conversely, over-expression of Mortalin had the opposite effect, with a decrease of shorter and an increase of longer mitochondria (38%, 39% and 23% for short, intermediate and long mitochondria, respectively). This increased filamentation of the mitochondrial network in neurons over-expressing Mortalin was kept upon rotenone treatment (Sup Fig. 6), suggesting a crucial role of Mortalin on mitochondrial morphology in degenerative neurons.

**Figure 5:**
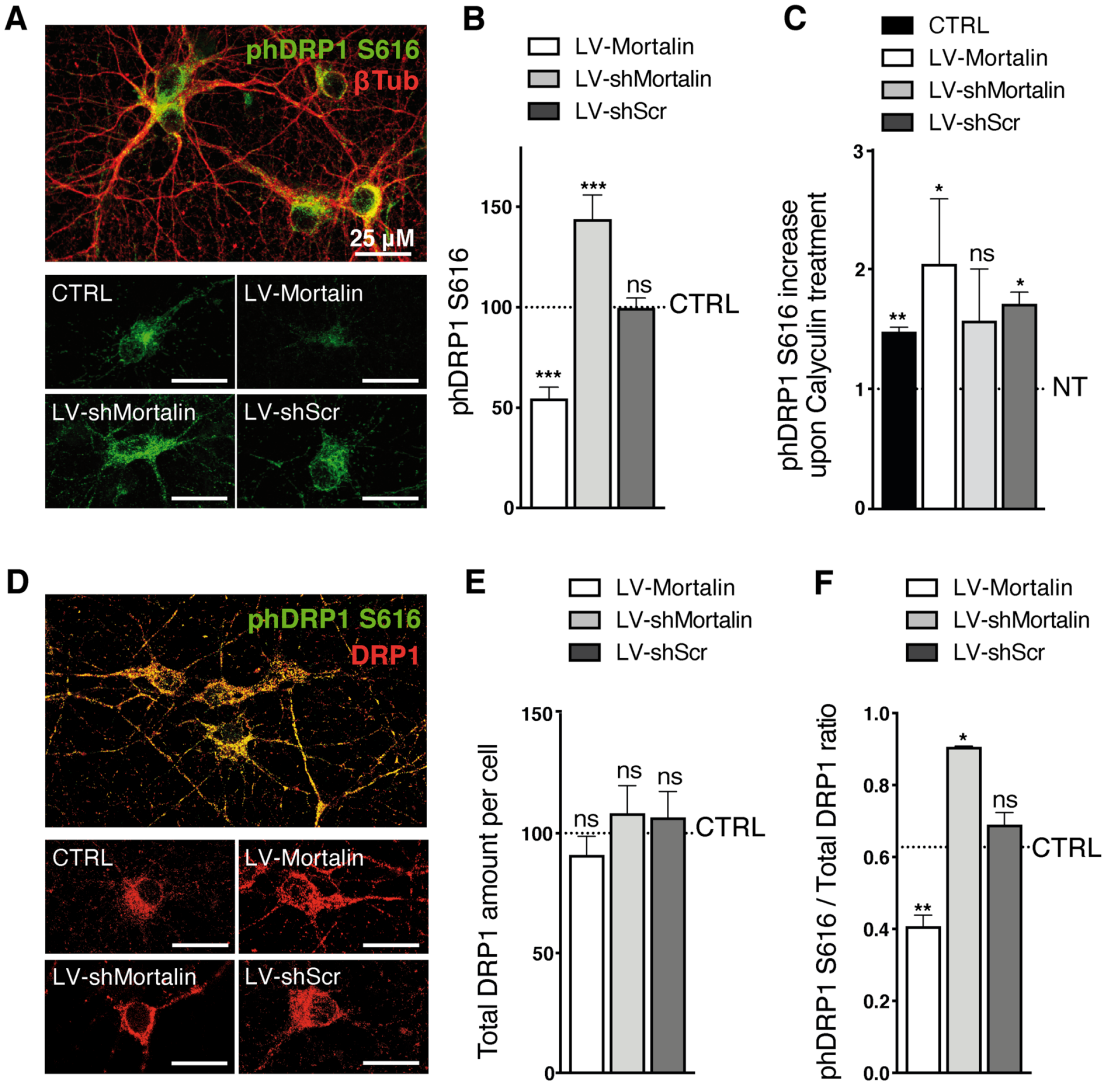
Mortalin impacts mitochondrial morphology by modulating Drp1-S^616^ phosphorylation. Rat embryonic primary cortical neurons were transduced on DIV 3 with lentiviral vectors (LV) to induce over- (LV-Mortalin) or under- (LV-shMortalin, clone **A**) expression of Mortalin. A lentiviral vector expressing a shRNA with a scramble sequence was used as a control (LV-shScr). Neurons were then fixed on DIV 12 and the active form of Drp1, phosphorylated on serine 616, was revealed by pDrp1-S^616^ staining. (**A**–**C**) The neuronal network was visualized with the neuronal marker βIII-tubulin (βIII-Tub). On each randomly taken picture (**A**), the total amount of pDrp1 was measured (integrated staining density, Image J software) and normalized on the βIII-tubulin staining area. For each experiment, 4 pictures were taken per condition and the corresponding mean ratios were normalized relative to control, non-transduced neurons. (**B**). The same was done upon treating neurons with Calyculin-A for 30 min before fixation, in order to inhibit phosphatase activity (**C**). The results are presented as means ± SD of 4 experiments. (**D–F**) Neurons were co-strained with antibodies directed against total Drp1 and pDrp1-S^616^ (**D**). For each randomly taken picture, we evaluated the total and phosphorylated protein amounts in βIII-Tub + cells, using the integrated staining density (Image J software). The values obtained for total Drp1 were normalized on the signal for non-transduced neurons in each experiment and are presented as means ± SD of 3 independent experiments (**E**). Ratios of the integrated staining densities for pDrp1-S^616^ on total Drp1 were also measured and normalized on the ratio obtained for un-transduced cells and presented as means ± SD of 3 independent experiments (**F**). **p < 0.01, by Mann–Whitney non parametric test.

Mitochondria morphology is controlled by the activity of different GTPases. Among them, Drp1 (Dynamin Related Protein 1) drives mitochondrial fission^40^. We thus wondered whether Mortalin control of mitochondrial elongation could result from changes in the molecular processes regulating mitochondrial Drp1 activity. In particular, the fission activity of Drp1 depends on its phosphorylation status, phosphorylation on serine 616 being associated with enhanced mitochondrial fission^41^. To determine the impact of Mortalin levels on pDrp1 S616, we measured the integrated densities of immunostaining for phosphorylated Drp1 in primary neurons (Fig. 5A). We observed that the S616 phosphorylated form of Drp1 was strongly reduced in neurons over-expressing Mortalin (-35% compared to control). Conversely, Drp1 S616 phosphorylation was enhanced (+ 25%) in neurons expressing reduced Mortalin levels (Fig. 5B). Treatment of neurons with a phosphatase inhibitor (Calyculin) served as the validation for the pDrp1 S616 antibody that we used (Fig. 5C). We performed the same analysis upon normalization for total Drp1 levels Fig. 5D). We thus verified that total Drp1 levels were similar in all cases (Fig. 5E), and that modulation of Mortalin indeed affected Drp1 phosphorylation (Fig. 5F). It has been reported that Drp1 phosphorylation at Ser616 can be mediated by ERK1/2^42^ and that Mortalin may negatively regulate ERK1/2 activity^43^. However, western-blot analyses of phosphorylated ERK1/2 did not reveal any impact of Mortalin modulation on pERK1/2 levels, at least in our model of primary neurons (Sup Figs. 7 and 8).

## Discussion

Although it has been documented that Mortalin levels are reduced in the degenerative brain of PD patients^24^, this observation has not been linked to date with any genetic risk with degenerative diseases^26,28^. Thus, the interplay between this mitochondrial chaperone and the neuronal degeneration process remains largely unknown. In the present work, we show that Mortalin expression levels in neurons are directly correlated with the control of mitochondrial stress, through the modulation of axonal vulnerability. These observations corroborate the link between the oxidative stress management mediated by Mortalin, already described in non-neuronal cells^22^, as well as its down-regulation in patients’ brains.

Mechanistically, we demonstrate that Mortalin over-expression is axoprotective and that this property correlates with its ability to increase mitochondrial length, by interfering with the activation of the mitochondrial fission protein Drp1. This observation is in line with the recent correlation between Mortalin up-regulation and inhibition of Fis1, a Drp1 mitochondrial receptor, upon antioxidant treatment of hepatocytes^44^. Our data also further document the fundamental importance of mitochondrial dynamics in axons at the early stages of neurodegeneration^6,7,10,45,46^. It should, however, be reminded that mitochondrial dynamics is also required for essential processes regulating cellular physiology, particularly in neurons, such as mitochondrial transport or quality control through mitophagy, as well as synaptic activity. More specifically, fission is thought to be an adaptive response to axonal injury and reduced mitochondria fission may impair mitochondrial targeting to the nerve terminals, thereby causing degeneration of specific neuronal populations^47,48^. Finally, although it is established that Mortalin over-expression is protective, its expression is not physiologically boosted in degenerative neurons. Hence, the modalities by which Mortalin-induced over-filamentation drives protection against axonal insults might underlie complex molecular processes that remain to be determined. Similarly, it would be important in the future to determine the molecular mechanisms whereby Mortalin regulates Drp1 phosphorylation. Many kinases have been shown to be involved in this process, including ERK1/2 in cancer cells^42,43^ and in iPSC^49^, but also CDK1^41^, CDK5^50^, cAMP^51^ or PKCdelta^52^, and their links with Mortalin levels are important leads to pursue in the future.

In degenerative neurons, mitochondrial functional and morphological impairments are associated with increased levels of ROS, inducing an oxidative stress situation that affects nucleic acid stability, protein homeostasis and, ultimately, the functionality of ion channels and cellular defenses^53,54^. These events might be of critical importance in the progress of neurodegenerative pathologies. Mortalin was identified as one of the most oxidation-sensitive cellular proteins^55^. Under conditions of oxidative stress, Mortalin is able to activate cellular antioxidant processes, mainly through sequestration of DJ-1 within mitochondria^16^. Moreover, Mortalin is found in multiple subcellular localizations such as the endoplasmic reticulum (ER), cytosol, cytoplasmic vesicles and mitochondria^56^, where it interferes with different cellular pathways through binding to a wide range of specific protein partners (for review^57^). The majority of cellular Mortalin is, however, located within the mitochondrial matrix^13^.

Finally, Mortalin—rather known as Grp75 for this function—also plays a crucial role by tethering mitochondria and ER, by connecting proteins from the ER membrane (IP3R) and the mitochondrial outer membrane (VDAC) at contact membrane locations called MAMs (mitochondria-associated membranes). MAMs structure and function are altered in several neurodegenerative pathologies such as PD, concomitantly with Mortalin down-regulation^58^. Interestingly, MAMs functions and mitochondrial dynamics are closely related. In particular, Drp1-dependent mitochondrial fission occurs preferentially in the proximity of ER-mitochondria contacts^59,60^, while Ca^2+^ released from the MAMs activates Drp1, thereby causing mitochondria fission^61^. Hence, our observations regarding the impact of Mortalin on Drp1activation may further enlighten the decisive crosstalk between ER and mitochondria for proper management of stress, or—in case of prolonged or strong stress conditions—the initiation of neurodegeneration. To date, however, the involvement of MAMs in the axonal compartment and their precise role during the early stages of degeneration remains unclear. Further examination of the interplay between mitochondrial dynamics and ER-mitochondria crosstalk in the axonal compartment may provide new insights for a better understanding of the degenerative process and, hopefully, for the development of efficient therapeutics against neurodegeneration.

## Methods

### Primary cultures of cortical neurons

Pregnant Sprague–Dawley female rats were purchased from Janvier Labs. Prior to sacrifice, housing and care of rats was performed in a registered facility (agreement number: E3155508, renewed on Oct 24, 2019), in accordance with the European Union Council Directive 86/609/EEC. Our primary neuronal protocol received approval from the local ministry-approved committee on the ethics of animal experiments (“*Comité d’Ethique en Expérimentation Animale”*, CEEA-122 for "*Comité d’Ethique de l’US 006/CREFRE*"; permit number: PI-U1043-DD-10). This committee ensures that protocols respect the French National Charter for ethics on animal experimentation. As such, our study was therefore also performed in compliance with the ARRIVE guidelines (https://arriveguidelines.org/). Primary cortical neurons were isolated from embryos at gestational day 17 by the papain dissociation method, as described previously^9,10^. Before seeding, supports were coated with 500 µg/ml Poly-L-ornithine (Sigma), followed by 5 µg/ml laminin (Roche). Neurons were maintained in Neurobasal medium (Gibco) supplemented with 100 µg/ml penicillin/streptomycin, 2 mM glutamine, 2% B-27 supplement (Gibco). Depending on the experiment, neurons were seeded in 24-wells plates, either directly (fluorometric analyses) or on 12 mm diameter glass coverslips for immunofluorescence (100,000 cells/well), in 60 mm plastic culture dishes for biochemistry (1,5.10^6^ cells/dish) or in microfluidic-based culture devices.

Microfluidic chips were prepared as described before^6,9,10,39^, by molding polydimethylsiloxane (Sylgard 184, Dow Corning) into resin SU-8 silicon masters having a complementary positive pattern of the cell culture compartments and microchannels. Polymer prints and glass coverslips were treated for 1 min with an air plasma generator, then both elements were bonded together and coated with Poly-L-ornithine and laminin. Primary neuronal cell suspensions were seeded in the somatic chambers by introducing 3 μl of a 5 × 10^7^ cells/ml suspension in the upper reservoir, before filling both reservoirs with supplemented neuronal culture medium. A 10 μl differential medium volume was maintained between the somatic and axonal chambers, to ensure a permanent hydrostatic flux. Neurons were transduced with lentivectors (LV) 3 days after plating.

### Construction and Production of LV

To overexpress Mortalin, the coding sequence of rat Mortalin was obtained by extracting mRNAs from neurons, followed by specific reverse transcription and PCR amplification. The PCR product was sequenced and inserted into the pTrip vector (kind gift from P. Charneau, Pasteur Institute, Paris) using *Bam*HI and *Xho*I restriction sites, downstream of the constitutive cytomegalovirus enhancer/chicken β-actin (CAG) promoter. To downregulate Mortalin expression, LV plasmids driving the expression of a set of four 29-mer short hairpin RNAs (shRNA) specific for Mortalin, or a scramble control sequence, together with GFP were purchased from Origene (CAT# TL704140). All LVs were produced and purified as described previously^9,10^. Briefly, 10 T150 flasks were plated with 1.2 × 10^7^ HEK-293 T cells each, to reach 80% confluence the following day. Then, HEK-293 T cells were transfected with the packaging plasmids psPAX2 and pMD2.G (both obtained from Addgene), together with LV genomic plasmid (respectively, 14.6 μg, 7.9 μg, and 22.5 μg of endotoxin-free prepared plasmids mixed with 100 μL of GeneJuice (Merck) for each T150). Media containing lentiviral particles were harvested in serum-free OptiMEM (Gibco) 48 h later, cleared by low-speed centrifugation (150 g, 5 min at 4 °C), and filtered through 45-μm capped syringe filters. LVs were lastly purified by ultracentrifugation through a 20% sucrose cushion 80,000 g, 2 h, 4 °C; SW32Ti rotor; Beckman Coulter) and lentiviral pellets were resuspended in ice-cold PBS, pooled, and aliquoted in 10 μL samples stored at − 80 °C. LV concentrations were systematically evaluated after transduction of HEK-293 T cells with serial dilutions, by quantification of Mortalin levels by Western blot. LV-shRNA titers were also determined by counting GFP foci 72 h after transduction. We then used the LV suspension amounts necessary to either double or decrease by 50% the expression levels of Mortalin, relative to endogenous levels in non-transduced neurons.

### Treatments of primary cortical neurons

Neuronal cultures at 12 days in vitro (DIV12) were subjected to mitochondrial stress by adding different concentrations of the respiratory chain complex I inhibitor rotenone (Sigma–Aldrich), diluted in DMEM containing 1 g/L glucose (Gibco), supplemented with 2 mM glutamine, 1% penicillin/streptomycin, 2% B-27, 1% N2 supplements (Gibco). Carbonyl cyanide-4-(trifluoromethoxy)phenylhydrazone (FCCP, Sigma–Aldrich, 100 μM, 4 h) was used as a positive control of mitochondrial respiration failure, while treatments with N-acetylcysteine (NAC, Sigma–Aldrich, 100 μM, 24 h) and hydrogen peroxide (H_2_O_2_, Sigma–Aldrich, 100 μM, 30 min-2 h) were used to modulated ROS amounts. To inhibit the protease activity, carbobenzoxy-Leu-Leu-leucinal (MG132, Sigma Aldrich, 10 µM), was added for 4 or 24 h, according to rotenone treatment duration.

### Measurements of mitochondrial ROS

Mitochondrial ROS were measured using the MitoSOX Red Mitochondrial Superoxide Indicator (ThermoFisher), according to the manufacturer indications. In brief, cells were washed in Hank's Balanced Salt Solution (HBSS, Gibco), and then incubated 10 min with 5 μM of MitoSOX dye diluted in HBSS. After two HBSS washes, fluorescence intensities of the cells were measured using a Varioskan apparatus (Flash Multimode Reader, Thermo Scientific, Em 510 mm/Ex580 mm). All measures were made twice with biological duplicates, and the value obtained with the diluted MitoSOX dye without cells was subtracted to all mean values.

### Mitochondria isolation for Western blotting

We performed sub-cellular fractionation to obtain mitochondria-enriched fractions. To this aim, 2.10^7^ rat primary cortical neurons were pelleted, rinsed twice in PBS and resuspended in homogenization buffer (HB: 10 mM Tris–HCl, pH7.5, 2 mM MgCl2, 10 mM KCl, 250 mM Sucrose, Protease Inhibitor cocktail, 0.5 mM DTT). Neurons were then frozen at – 80 °C and thawed, transferred to a 2 ml Potter homogenizer, triturated for 20 times and further centrifuged at 1,000 g for 10 min. The supernatant was then centrifuged at 12,000 g for 15 min, afterwards the pellet, containing mitochondria, was resuspended in HB + 1% Triton X-100 for protein quantification by Western Blot.

### Western blotting

For protein quantification, we proceeded as described before^62^. Indeed, protein concentrations were determined with a standard Bradford assay and samples were boiled for 10 min in Laemmli buffer. A total of 15 μg of samples were loaded on 4–15% Tris–glycine gels (Bio-Rad), run 1 h at 100 V, transferred onto nitrocellulose membranes and blocked for 1 h in Odyssey blocking buffer diluted 1:1 in Tris buffer saline (TBS). After blocking, membranes were incubated with primary antibodies diluted in blocking buffer, overnight at 4 °C [rabbit polyclonal anti-Mortalin (Origene Technologies, 1/5,000); mouse monoclonal anti–β-actin, (Sigma–Aldrich, 1/100,000); mouse monoclonal anti-total ERK1/2 (Cell Signaling, 1/2,000) and rabbit anti-ERK1/2 phThr202/Tyr204 (Cell Signaling, 1/2,000); mouse monoclonal anti-Hsp60 antibody (Santa Cruz Biotechnology, 1/1000)]. Membranes were then washed extensively in Tween-20 containing TBS (TBST) and incubated for 1 h at RT with secondary antibodies: goat anti-rabbit Alexa 680 and goat anti-mouse Alexa 770 (Biotium, 1/15,000) both diluted in Odyssey blocking buffer (1:1 v/v TBS). Laser scanning and analyses of the blots were performed using the Odyssey Infrared Imaging System (Li-Cor).

### Immunofluorescence and imaging

Neurons were fixed with PBS containing 4% paraformaldehyde preheated to 37 °C, for 20 min at room temperature before being stained as described earlier^62^. To this aim, cells were then permeabilized with PBS containing 0.25% Triton X-100 for 5 min, rinsed twice with PBS, and blocked for at least 1 h with PBS containing 2.5% normal goat serum and 3% BSA. The cells were subsequently incubated for 2 h at 37 °C with primary antibodies diluted in blocking solution [anti-βIII-tubulin (Sigma–Aldrich, 1:500); anti-translocase of outer mitochondrial membrane 20 (Tom20, Santa Cruz Biotechnology, 1:500), anti-cyclin B1 (Thermo Fisher Scientific clone V152, 1:200), anti-phospho-DRP1^S616^ (Cell Signaling Technologies, 1:1,000)], rinsed twice for 10 min each in PBS containing 0.1% Triton X-100 and once in PBS, and then incubated 45 min at room temperature with Alexa-conjugated fluorescent secondary antibodies (Sigma–Aldrich, 1:500 dilution in PBS). After several washes in PBS, the cells were mounted in Vectashield medium containing DAPI (Vector Laboratories). All microscopy analyses and measurements were performed on an inverted SP8 confocal microscope (Leica Microsystems) fitted with × 20 (microfluidics) or × 63 (mitochondria and DRP1) objectives. For pyknosis analysis, the DAPI staining was considered in βIII-tubulin^+^ cells. For each culture, a minimum of 100 nuclei were observed and assessed as healthy or pyknotic according to their morphology. For phospho-DRP1^S616^ quantifications, whole-cell fluorescence intensity was measured in βIII-tubulin^+^ cells, using the integrative fluorescence intensity tool of the ImageJ software (National Institutes of Health, Bethesda, MD, USA). To further validate our phospho-DRP1^S616^ antibody, we also treated cells with the phosphatase inhibitor Calyculin-A (Sigma–Aldrich, 30 min, 10 nM).

### Induction of oxidative stress

Neuronal cultures at 12 days in vitro (DIV12) were subjected to oxidative stress by adding different concentrations of the respiratory chain complex I inhibitor rotenone (Sigma–Aldrich), diluted in DMEM containing 1 g/L glucose (Gibco), supplemented with 2 mM glutamine, 1% penicillin/streptomycin, 2% B-27, 1% N2 supplements (Gibco). Carbonyl cyanide-4-(trifluoromethoxy)phenylhydrazone (FCCP, Sigma, 100 μM) was used as a positive control of mitochondrial respiration failure.

### Analysis of axonal fragmentation of primary neurons in microfluidic-based culture devices

To assess the specific impact of oxidative stress on axons, we evaluated axonal fragmentation as previously described^9,10^. Briefly, rotenone (1 μM) diluted in low-glucose medium was applied in the somatic or axonal chambers (for, respectively, somatic or axonal stress) of neuronal cultures (DIV12), for 16 h. Neurons were then fixed and stained for βIII-Tubulin. The minor fraction of cultures displaying more than 25% of pyknotic nuclei was discarded. The numbers of Tubulin spots were counted in 4 randomly-chosen fields in each axonal chamber using Imaris software (Bitplane), while total βIII-Tubulin staining was assessed using the ImageJ software for each picture, thereby defining a fragmentation index. Staining with DAPI was systematically performed in the somatic chambers to check for nuclear integrity and ensure that the hydrostatic flux had been maintained to prevent leakage throughout axonal rotenone treatment.

### Statistical analyses

Results are presented as means ± SD. Data were analyzed by Mann–Whitney or 1-way/2-way ANOVA using *U*, Sidak’s or Kruskal Wallis and Dunn’s multiple comparisons as non-parametric post hoc tests with Prism software (GraphPad).

## Supplementary Information

Supplementary Information 1.
