# Supplementary material for: HSPA9/Mortalin mediates axo-protection and modulates mitochondrial dynamics in neurons
Source: Sci Rep. 2021 Sep 6;11:17705. doi: 10.1038/s41598-021-97162-1 (PMC8421332; doi:10.1038/s41598-021-97162-1)
Supplement: Supplementary file 1 — Supplementary Information 1. [file 41598_2021_97162_MOESM1_ESM.pdf]

**SUPPLEMENTARY INFORMATION**

# HSPA9/MORTALIN MEDIATES AXO-PROTECTION AND MODULATES MITOCHONDRIAL DYNAMICS IN NEURONS

C. Ferré[1#], A. Thouard[1#], A. Bétourné[1], A.L. Le Dorze[1], P. Belenguer[2], MC. Miquel[2], J.M. Peyrin[3], D. Gonzalez-Dunia[1*] and M. Szelechowski[1*]

[1]Toulouse Institute for Infectious and Inflamatory diseases (Infinity), Université Toulouse, CNRS, Inserm, UPS, Toulouse, France; [2]Centre de Recherches sur la Cognition Animale (CRCA), Centre de Biologie Intégrative (CBI), Université de Toulouse, CNRS, UPS, Toulouse, France; [3] Sorbonne Universités, Faculté des Sciences et Technologie, CNRS UMR 8246, INSERM U1130, Neurosciences Paris Seine, Institut de Biologie Paris Seine, Paris, France.

# Joint first authorship

* Corresponding authors (daniel.dunia@inserm.fr or marion.szelechowski@inserm.fr)

## SUPPLEMENTAL METHOD

### Measure of mitochondrial membrane potential.

To assess the impact of rotenone and FCCP treatments on mitochondrial respiratory function, neurons were stained with JC-1 dye (2 µM in HBSS; Abcam) following recommendations of the manufacturer. JC-1 mitochondrial aggregation was evaluated through the quantification of fluorescence emission at 530 nm (green) and 590 nm (red), following an excitation at 485 nm using a VarioSkan apparatus (Flash Multimode Reader, Thermo Scientific). The red-to-green ratio was used as an indicator of the mitochondrial membrane potential ($\Delta\Psi m$).

## SUPPLEMENTAL FIGURES

**Sup Figure 1**

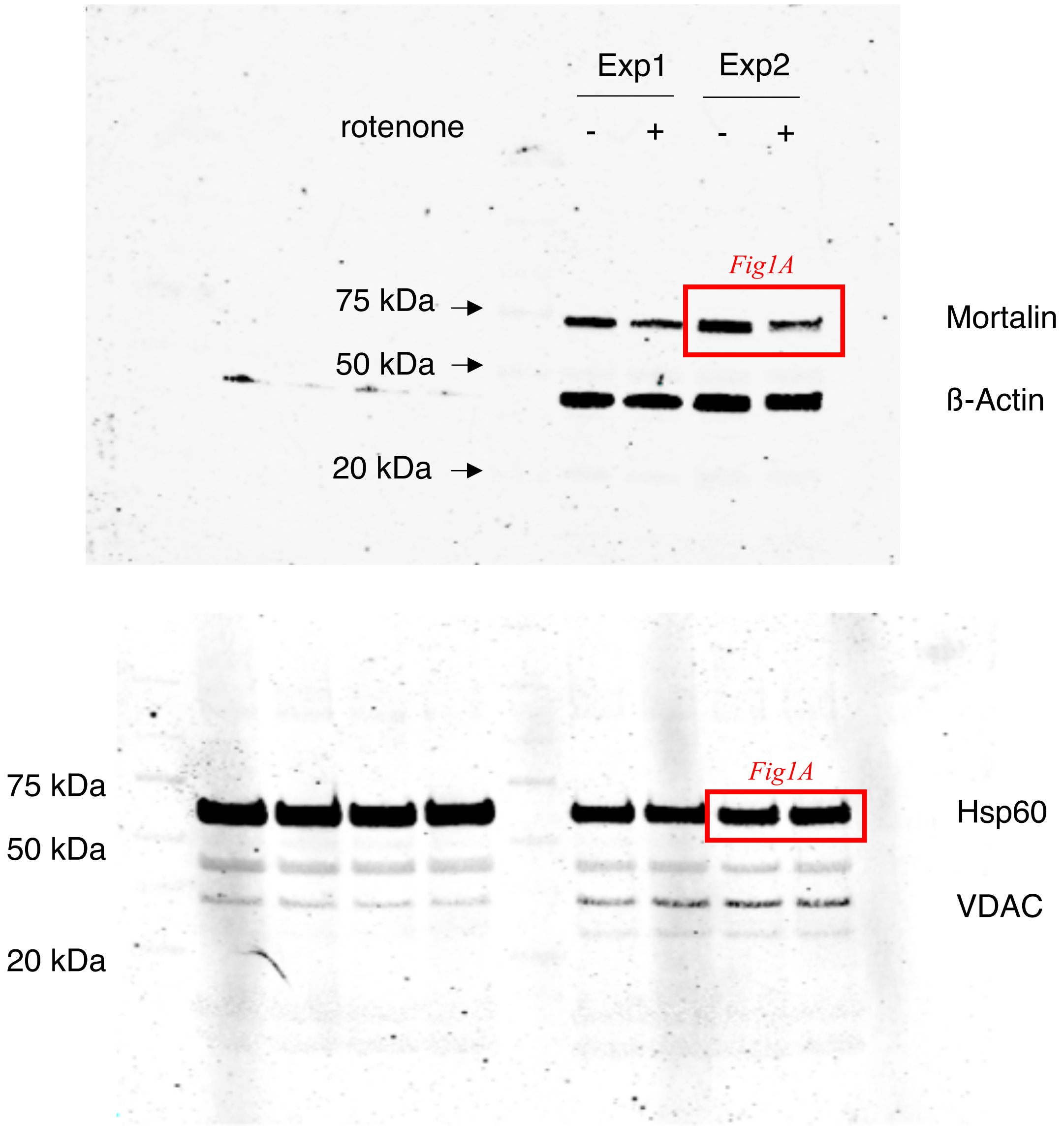


**Quantification of Mortalin in mitochondria-enriched fractions of cortical neurons with (+) or without (-) rotenone treatment (10nM, 4h). (Full scan of blots used for Fig1)**

ß-Actin together with the mitochondrial chaperone Hsp60 were used as loading controls. Staining was performed on 2 different blots because Mortalin and Hsp60 migrate close to each other. The blot stained with Hsp60 antibody was also stained with a VDAC antibody that was not analyzed in this study. Molecular weight markers are pointed out for each blot. The elements used for Figure 1A are shown as red boxes.

<u>**Sup Figure 2**</u>

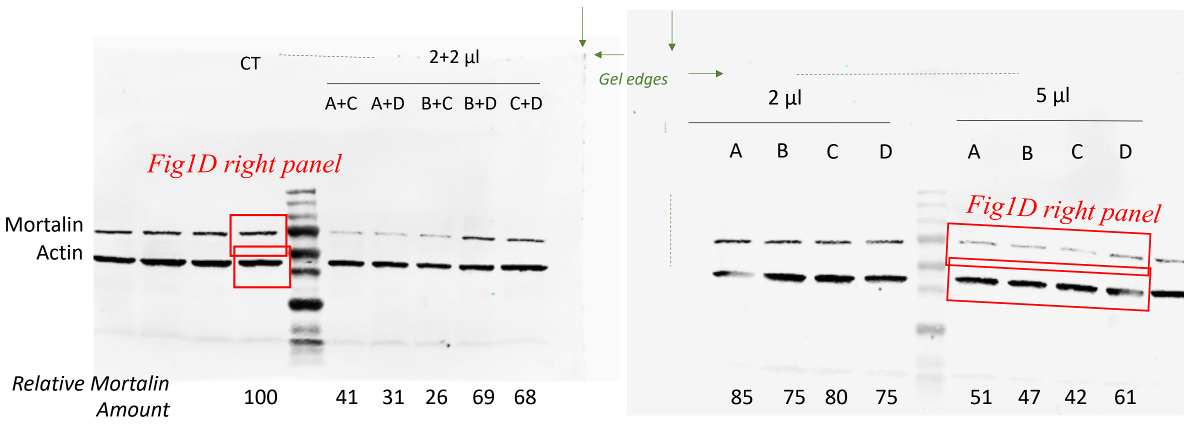


**Comparison of Mortalin protein down-regulation using 4 distinct shRNA sequences directed against Mortalin, alone or in combination. (Full scans of blots used for Fig 1).** Four lentiviral vectors expressing 4 different shRNA were compared for their abilities to down-regulate Mortalin protein amounts. ß-Actin was used as a loading control. The figure shows the full area that was scanned using the Odyssey Imager. In red boxes are presented the parts of the blots used for Main Figure 1D (right panels), in green (arrows and dotted lines) are indicated and/or drawn the gel edges.

**Sup Figure 3**

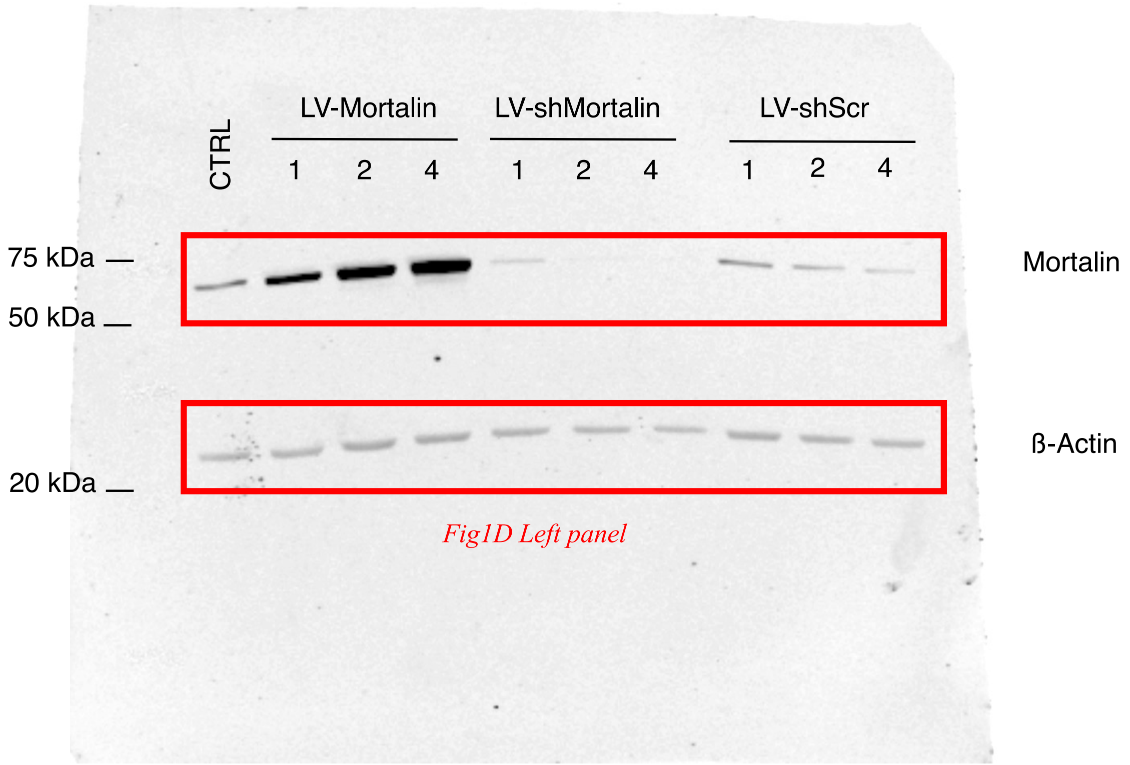


**Full scan of blots of Mortalin quantification (used for Figure 1) after lentiviral vector-driven over-expression (LV-Mortalin) or down-regulation (LV-shMortalin), using different MOI (1,2,4).** A lentiviral vector expressing a scramble sequence of shMortalin (LV-shScr) was used as a control of RNA interference. ß-Actin was used as a loading control. Molecular weight markers that were only visible on the membrane are pointed out. In red boxes are presented the parts of the blot used for Main Figure 1D (left panel).

**Sup Figure 4**

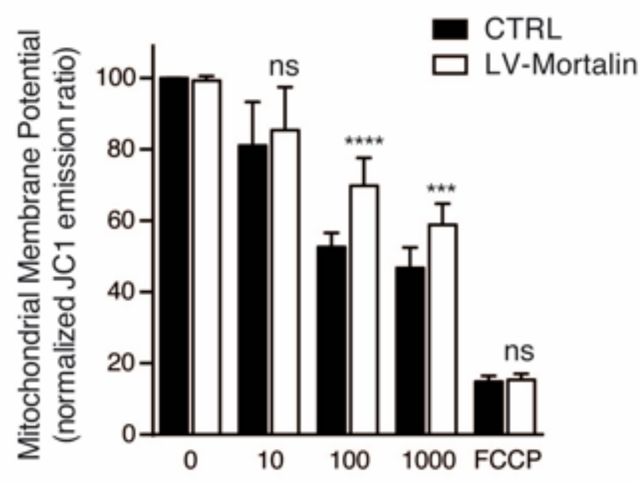


**Mortalin overexpression allows to maintain mitochondrial membrane potential upon rotenone exposure.**

Rat embryonic primary cortical neurons were transduced on DIV 3 with lentiviral vectors (LV) to induce overexpression of Mortalin (LV-Mortalin). Neurons were then treated or not with rotenone at 10, 100 or 1000 nM for 4 hours, and the mitochondrial membrane potential was assessed by JC-1 staining, followed by measurements of red-to-green fluorescence ratios. These ratios were normalized to control, non-treated neurons. Each experimental case was performed in triplicate and results are presented as means ± SD of 3 independent experiments.

*** $p<0.01$, **** $p<0.001$, by Mann-Whitney non parametric t-test.

## Sup Figure 5

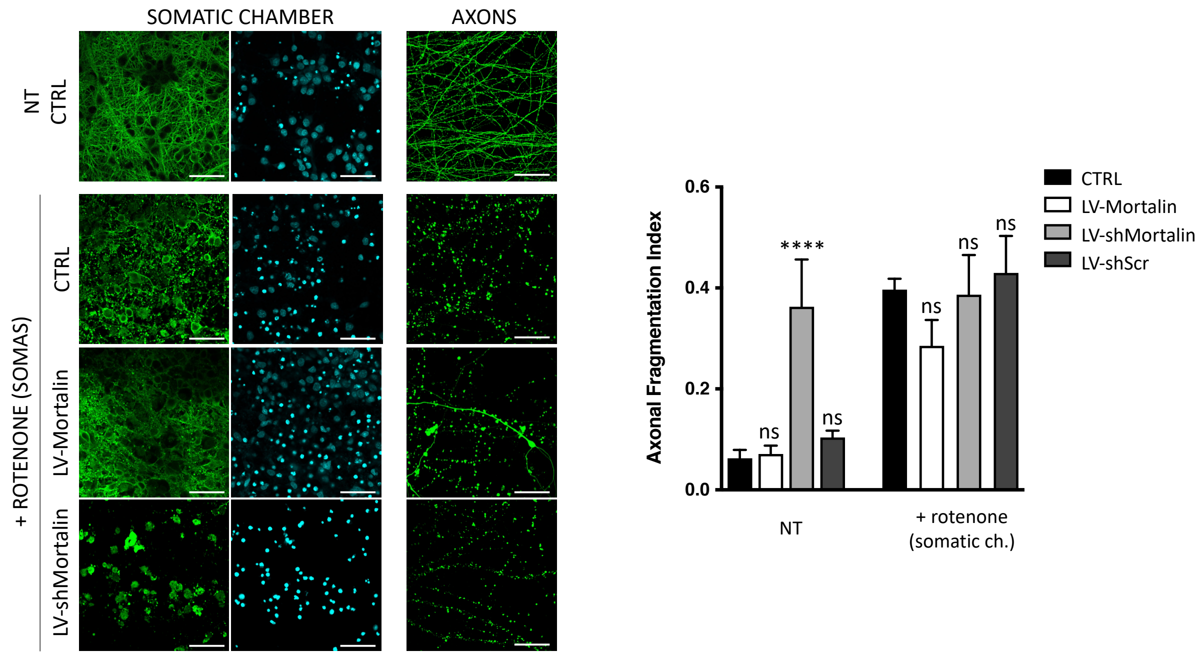


**Somatic treatment with rotenone leads to strong axonal fragmentation, regardless of Mortalin expression levels.**

Rat embryonic primary cortical neurons were grown in microfluidic devices and were transduced on DIV 3 with lentiviral vectors (LV) to induce over- (LV-Mortalin) or under- (LV-shMortalin) expression of Mortalin. A lentiviral vector expressing a shRNA with scramble nucleotide sequence was used as a control (LV-shScr). On DIV 11, neurons were treated or not with 1 µM rotenone applied in the somatic chamber (+ rotenone) for 16 hours. Left: Pictures of βIII-tubulin staining were randomly taken within somatic and axonal chambers (4 by condition) and the axonal fragmentation indexes were measured for each picture as the number of axonal dots per unit of tubulin staining. DAPI staining was used to detect pycnotic nuclei. Right: Results are presented as means ± SD of 6 to 8 independent microfluidic devices, from 4 independent neuronal cultures. **** $p<10^{-4}$, by 1-way ANOVA with Sidak's multiple comparison *post-hoc* test.

**Sup Figure 6**

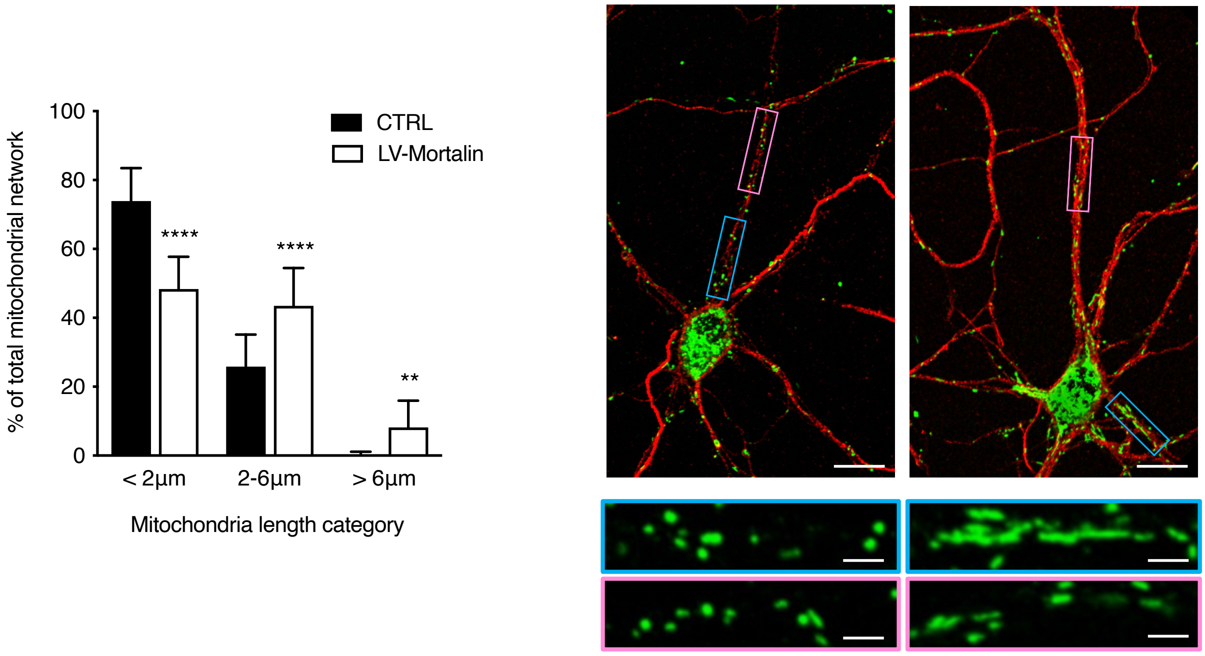


**Mortalin overexpression allows to maintain mitochondrial morphology upon exposure to rotenone.**

Rat embryonic primary cortical neurons were transduced (or not) on DIV 3 with lentiviral vectors (LV) to induce over-expression of Mortalin. On DIV 12, neurons were treated with 10 nM rotenone for 4 hours, fixed and both neuronal and mitochondrial networks were visualized by staining with, respectively, Tom20 and βIII-tubulin (βIII-Tub). Pictures were randomly taken and mitochondrial lengths were measured in all neural extensions. For each neuron, the sum of the lengths of all mitochondria was calculated (mitochondrial network), within which the relative proportions of short (<2µm), medium (2-6µm) and long (>6µm) mitochondria were considered. The graph represents means ± SD of 23 (Ctrl) and 28 (LV-Mortalin) neuronal mitochondrial networks from 3 independent neuronal preparations.

** p<0.1, **** p<0.001, by Mann-Whitney non parametric t-test.

**Sup Figure 7**

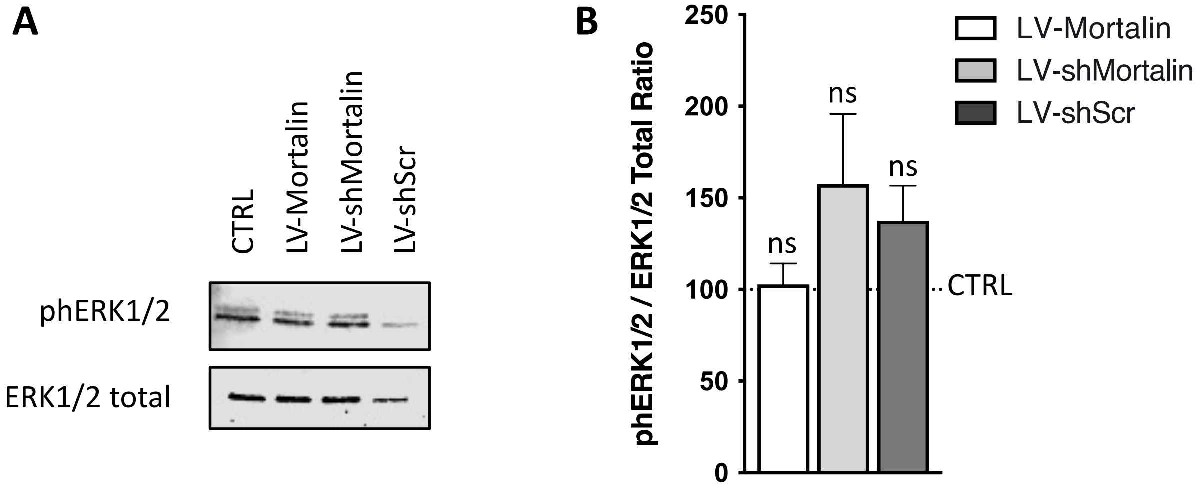


**Mortalin expression levels does not impact ERK1/2 phosphorylation levels.**

(A) Phosphorylated ERK1/2 (Left panel) or total ERK ½ (Right panel) were analyzed by Western blotting in primary cortical neurons over- or under-expressing Mortalin (LV-Mortalin or LV-shMortalin, respectively) after 12 days of culture. (B) Graph representing the ratio of phosphorylated ERK1/2 on total ERK1/2 signals, as means ± SD of 3 independent experiments. For each experiment, ratios were normalized on the ratios obtained for non-transduced neurons (CTRL). Mean ratios were compared to CTRL using Mann-Whitney non parametric test.

**Sup Figure 8**

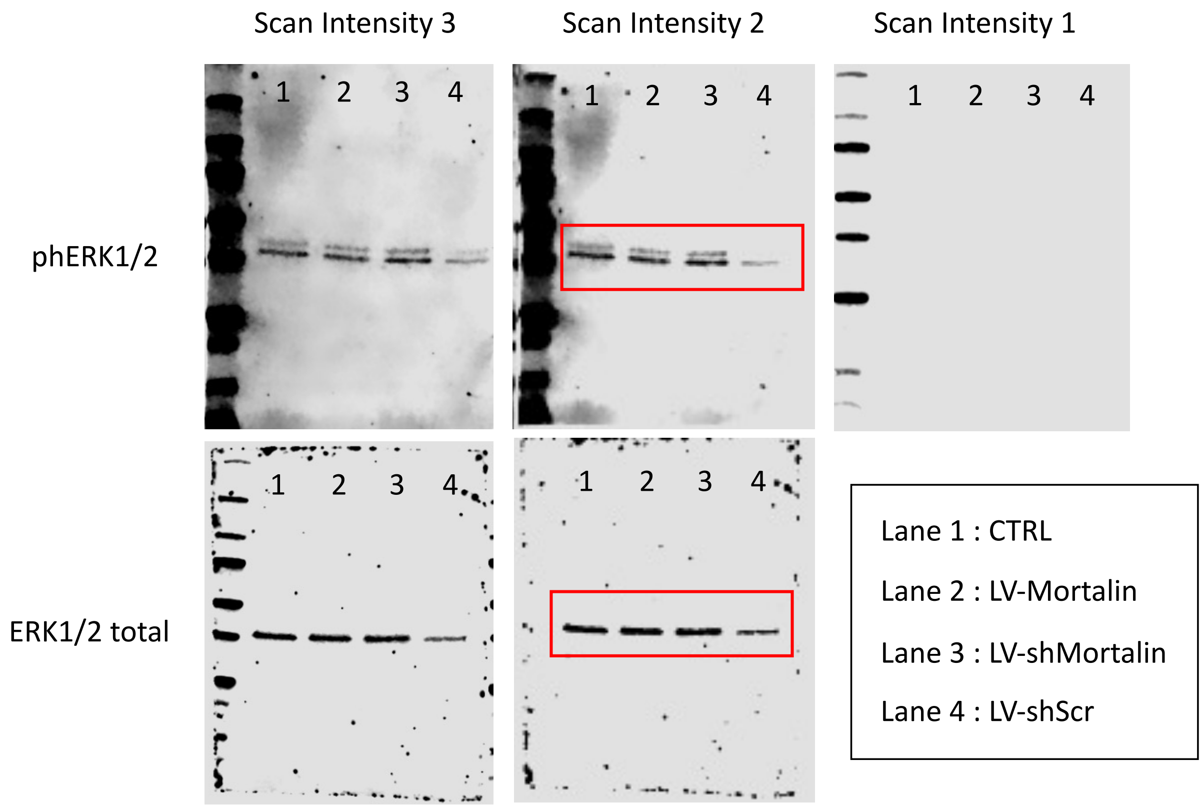


**Full scans of blots detecting phosphorylated and total ERK1/2, with different intensities.**

The red boxes show areas used for Sup Figure 7
